# Pre- and Post-Operative Nutrition Assessment in Patients with Colon Cancer Undergoing Ileostomy

**DOI:** 10.3390/ijerph17176124

**Published:** 2020-08-23

**Authors:** Georgios Vasilopoulos, Panagiota Makrigianni, Maria Polikandrioti, Ilias Tsiampouris, Dimitrios Karayiannis, Nikoletta Margari, Lamprini Avramopoulou, Georgia Toulia, Georgia Fasoi

**Affiliations:** 1 Department of Nursing, University of West Attica, 12243 Athens, Greece; gvasilop@uniwa.gr (G.V.); mpolyk@uniwa.gr (M.P.); iliastsiampour@yahoo.gr (I.T.); nmargari@uniwa.gr (N.M.); gtoylia@uniwa.gr (G.T.); gfasoi@uniwa.gr (G.F.); 2 Department of Clinical Nutrition, Evangelismos General Hospital, 11521 Athens, Greece; dkarag@hua.gr; 3 Intensive Care Unit, G. Gennimatas General Hospital, 11527 Athens, Greece; lambavra@yahoo.gr

**Keywords:** ileostomy, nutrition assessment, risk of malnutrition, weight loss

## Abstract

Introduction: Patients undergoing ileostomy surgery often experience electrolyte disturbances and dehydration, especially during the first post-operative period. Recently, research has also begun on how the newly constructed ileostomy affects the patient’s nutritional status. Aim: The aim of the present pilot study was to assess the nutritional status of patients before and after the construction of the ileostomy as well as nutrition-related factors. Material and Method: This was a pilot study. The sample consisted of 13 adult patients diagnosed with colorectal or colon cancer who underwent scheduled ileostomy surgery. The evaluation tool used was “Original Full Mini Nutritional Assessment (MNA)”. Patients underwent nutritional assessment before the surgery (time 0), on the 7th post-operative day (time 1), and on the 20th post-operative day (time 2). The statistical significance level was set at *p* < 0.05. Results: All patients had a drop in MNA score on the 7th and 20th post-operative days. Factors associated with MNA were weight loss, mobility, body mass index (BMI), number of full meals consumed per day, portions of fruits and vegetables consumed per day, and mid-arm circumference, *p* < 0.05, respectively. Pre-operatively, 38.5%, of patients had severe weight loss (>3 kg), 23% moderate weight loss and 38.5% minimal weight loss. Pre-operatively, 92.3% of participants were able to move on their own and 69.2% on the 20th post-operatively day. Furthermore, BMI >23 kg/m^2^ had 84.6% of participants pre-operatively and 30.8% on the 20th post-operative day. In terms of portions of fruits and vegetables consumed per day, 30.8% of patients consumed at least 2 times, pre-operatively and no one (0%) on the 20th post-operative day. Moreover, pre-operatively all participants (100%) had arm circumference >22 cm while on the 20th post-operative day, only 38.5% of participants had arm circumference >22 cm. Conclusions: In the first 20 days after the construction of an ileostomy, the nutritional status of the patients is significantly affected. Decreased patient nutrition in both quantity and ingredients and reduced fluid intake appear to adversely affect the patient’s nutritional status.

## 1. Introduction

During recent decades, the number of ileostomies created has been expanding enormously due to surgical management of various intestinal disorders. Depending on indications, surgical technique and emergency demands, stomas may be either temporary or permanent [1]. An ileostomy is a surgically created opening of a piece of ileum on the abdomen through which digested food passes into an external system. The most prevalent causes that lead to ileostomy construction are bowel cancer, trauma and acute abdomen conditions [2].

An ileostomy is frequently associated with various stoma complications, reference [3,4] which occur in up to 50% of cases, [5] and are attributed to both operative and patient related factors [5,6,7]. Significantly more, ileostomy is related with increased morbidity, mortality and a staggering economic burden on patients and healthcare system [7].

In terms of clinical characteristics, an ileostomy is associated with malnutrition, excessive output (defined as output ≥1500 mL for two consecutive days) and problems related to leak or stoma appliances [8,9,10]. In more detail, the ileum is responsible for the absorption of lipids, carbohydrates, proteins and vitamin B12 [9]. Patients being typically deprived of their terminal ileum are at higher risk for dehydration, impaired nutritional status, electrolyte imbalances, deficiencies in B-12, iron, magnesium, fat, and folic acid [11,12]. Therefore, it is clinically meaningful to provide nutrition support (oral, enteral or parenteral), water and electrolytes in order to prevent malnutrition [9] along with proper dietary requirements [11].

Prompt evaluation of nutritional status allows the identification of patients at risk, thus contributing to the recovery after surgery. Also, this evaluation may improve clinicians’ ability to empower patients to manage their ileostomy more efficiently [6].

To the best of our knowledge, data exploring nutritional status of patients with an ileostomy are limited. However, it is widely accepted that nutritional evaluation is important with respect to patient outcomes.

Thus, the aim of this study was to explore the nutritional status of patients with an ileostomy in three periods of time: (a) before the surgery (time 0), (b) 7th post-operative day (time 1), and (c) 20th post-operative day (time 2) as well as to identify factors associated with nutrition status.

## 2. Method and Material

### 2.1. Study Population, Design, Setting, and Period of the Study

In the present pilot study were enrolled 13 adult patients diagnosed with colorectal cancer who underwent scheduled surgery for an ileostomy in a public hospital in Attica. All participants had Standard (Brooke) end ileostomy.

It was a convenience sample. The study included patients during the period August 2017–July 2018.

### 2.2. Sample: Inclusion and Exclusion Criteria

During the period which the research was conducted, from a total of 20 patients who were initially identified as eligible for participation, only 13 were finally enrolled because 7 refused to participate or had other co morbidities.

Inclusion criteria in the study were as following, patients: (a) being diagnosed with colorectal cancer (b) hospitalized in a public hospital in Athens during the study period and (c) having the ability to write and read the Greek language fluently.

Exclusion criteria were as following, patients: (a) with a history of mental illness, (b) patients with other inflammatory bowel disease (ulcerative colitis and Crohn’s disease) and (c) being unable to communicate throughout the study period.

### 2.3. Data Collection and Procedure

Collection of data was performed by the method of the interview using a questionnaire which was developed by the researchers so as to fully serve the purposes of the study. Completion of the each questionnaire lasted approximately 15 min and took place in the evening shift when patients were free of other tasks or examinations.

Also, data were collected through medical history or physical assessment, or in collaboration with other specialists.

Measuring height accurately, participants had to stand with feet flat, together, and against the wall. A metal tape was used to measure from the base on the floor to the marked measurement on the wall to get the height measurement. To measure weight accurately, a digital scale was used which was placed on a firm flooring while participants had to stand with both feet in the center of the scale. All patients underwent weight measurements under the same circumstances (the same scale, the same clothing, the same hour). Body mass index (*BMI*) calculation based on the following formula: BMI = body weight/height^2^ (kg/m^2^). BMI classification was adopted: <18.5 underweight, 18.5–24.9 normal body weight, 25.0–29.9 overweight, and <30.0 obesity.

Moreover, patients underwent nutritional assessment in three periods: (a) before the surgery (time 0), (b) 7th post-operative day (time 1), and (c) 20th post-operative day (time 2).

In the present study there was no intervention or control group since this research merely recorded nutritional status in patients with ileostomy before and after surgery.

### 2.4. Nutritional Assessment (Study Instrument)

To measure nutritional state, the Mini Nutritional Assessment (MNA) was used. This screening tool identifies persons who are malnourished or at risk of malnutrition. MNA which was developed almost 20 years ago, still remains the most widely used screening tool for malnutrition among adults or the elderly. Initially, this tool included 18 questions (Original Full MNA) while later was constructed the Short Form of MNA consisting of 6 questions to simplify the process [13,14,15,16].

In our pilot study to assess the nutrition of patients with ileostomy, it was used the Original Full MNA which is available at https://www.mna-elderly.com/ [13].

Original Full MNA is recommended for a more detailed assessment of patients’ nutritional status and apart from demographic data, it also includes clinical features, Specifically more it includes:

- decrease in food intake due to loss of appetite, digestive problems, chewing or swallowing difficulties, during the last 3 months;
- involuntary weight loss, during the last 3 months;
- mobility;
- psychological stress or acute disease, during the last 3 months;
- neuropsychological problems;
- body mass index (BMI);
- independently living (not in a nursing home);
- more than 3 prescription drugs per day;
- pressure sores or skin ulcers;
- meals per day;
- consumption of two or more servings of fruits or vegetables per day;
- consumption of fluid (water, juice, coffee, tea, milk);
- mode of feeding;
- self view of nutritional status;
- self-perceived health status;
- mid-arm circumference and calf circumference.

The final score attributed to the patient (malnutrition indicator score) ranged from 0–30. Patients with less than 17 points were characterized as “malnourished”, those with 17–23.5 points as “at risk of malnutrition” while patients with 24–30 points as “at normal nutritional status” [13,14,15].

MNA is a simple tool to measure nutritional status. MNA has been used in hundreds of studies and translated into more than 20 languages with high sensitivity, specificity, and reliability. MNA is recommended by many national and international clinical and scientific organizations and can be used by a variety of health professionals, including physicians, dietitians, nurses or research assistants [14].

MNA provide several advantages in patients with an ileostomy. More in detail, this short and valid tool which is easily applied in daily practice, may help clinicians to develop prompt strategies to improve the nutritional state of ileostomy patients. Furthermore, in patients with malnutrition, the perioperative support may decrease the risk of post-operative leakage and infectious complications [16].

Last but not least, MNA is widely used in patients with cancer of all ages even though it is neither developed specifically for this disease nor for persons younger than 65 years [17].

### 2.5. Ethical Considerations

The study was approved by the Thesis Review Committee of the Post-Graduate Program “Wound care and Treatment” of the Department of Nursing of the University of West Attica (Approval Reg Number 123 - 6/2/2018). Patients who met the entry criteria were informed by the researcher for the purposes of this research. All patients participated only after they had given their written consent. Data collection guaranteed anonymity and confidentiality. All subjects had been informed of their rights to refuse or discontinue participation in the study, according to the ethical standards of the Declaration of Helsinki (1989) of the World Medical Association.

Informed Consent: Informed consent was obtained from all individual participants included in the study.

### 2.6. Statistical Analysis

All statistical analyzes were performed with the SPSS statistical package (IBM SPSS Statistics, version 21.0, Armonk, NY, USA, IBM Corp.). The regularity of the distributions of continuous quantitative variables was assessed by the Shapiro–Wilk criterion, as well as by the use of graphs to control symmetry and curvature (*P*-*P* or Q-Q plots).

Continuous variables were expressed as intermediate values (25th–75th percentile) and qualitative-categorical variables as absolute numbers and relative frequencies (%).

We checked the statistical significance of differences between groups with Mann–Whitney U and Kruskal–Wallis tests for variables that did not follow the normal distribution.

All statistical value values (*p*-values) emerged from bilateral tests and set at a statistical significance level of 5% for all analyses.

## 3. Results

The study population consisted of 13 patients, 10 men (76.9%) and 3 women (23.1%) who had colorectal cancer and underwent ileostomy surgery. Patients’ characteristics are shown in Table 1.

In terms of MNA score, the median score in time 0 (before surgery) was 24, in time 1 (7th day post-surgery) was 18.5 and in time 2 (20th day post-surgery) was 19.0.

According to MNA scale, ranges from 24 to 30 are characterized as “at normal nutritional status”. Therefore in time 0, patients are at normal nutritional status whereas in time 1 and in time 2, patients are at “risk of malnutrition” since values range from 17–23.5 points are characterized as at risk. Mini Nutritional Assessment scores range values are shown in Table 2.

**Figure 1:**
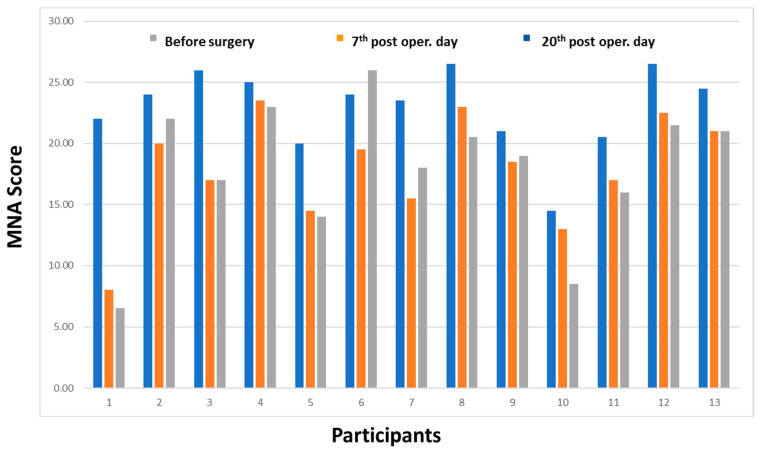
MNA scores in 3 times.

Figure 1 shows values of MNA in all periods (time 0, 1, 2). All patients had a drop in MNA score, except in the case of a patient (subject 6 in this chart). The score decreased more in the patient with the number 1.

Table 3 presents factors associated with MNA score. More in detail, factors that were statistically significantly associated with time 0, 1, 2 (pre-operative-7th post-operative-20th post-operative day) were as follows:

(a) Weight loss. Pre-operatively, 38.5% of patients had severe weight loss (>3 kg), 23.1% had moderate weight loss and 38.5% had minimal weight loss. Respectively, the percentages on the 7th post-operative day were 46.2%, 15.4% and 38.5%, while on the 20th post-operative day 53.8%, 15.4% and 30.8%. The difference in the percentage of those who suffered total weight loss on the 7th and 20th day, was statistically significant (*p* < 0.05).

(b) Mobility. A statistically significant difference was observed between individuals who pre-operatively could move on their own and those in the 20th post-operative day, (92.3% vs. 69.2%), *p* < 0.05.

(c) Body Mass Index. Pre-operatively, 84.6% of participants had BMI >23 kg/m^2^ while after the 20th post-operative day, 30.8% of participants had BMI >23 kg/m^2^, *p* < 0.05.

(d) The number of full meals consumed per day. Pre-operatively, 84.6% of participants had at least 2 meals per day, while on the 20th day, 69.2% of participants had at least 2 meals per day, *p* < 0.05.

(e) The portions of fruits and vegetables consumed per day. Pre-operatively 30.8% of patients consumed at least 2 servings of vegetables and fruits per day while on the 20th day, no one (0%) consumed 2 servings of fruits and vegetables, *p* < 0.05.

(f) The mid-arm circumference. Pre-operatively all patients had an arm circumference of more than 22 cm, while on the 20th post-operative day only 38.5% had an arm circumference >22 cm, *p* < 0.05.

## 4. Discussion

This pilot study explored the nutritional state of 13 patients who underwent an ileostomy due to colon cancer.

In terms of demographic characteristics, participants’ age ranged from 52.5 to 71 years. The prevalent age for colorectal cancer was over 50 years, however a rise in younger individuals was noticed, thus supporting the need for colonoscopy screening at the age 45 in order to detect those with early-onset [18,19,20]. Haleshappa et al. [19] showed that 27.8% of 89 patients were diagnosed with colon cancer in the age <40 years. More awareness to young-onset will be critical to improve outcomes in this patient population [20].

In terms of sex, men are at a slightly higher risk of developing colon cancer than women. Worldwide, colorectal cancer is the third most common cancer while for women, rectal cancer does not figure in the top 10 cancers, whereas colon cancer ranks 9th [19,20].

This pilot study showed a weight loss and reduction in BMI from pre-operative measurement to 3rd post-operative. More in detail, on the 20th post-operative day, 53.8% of patients had severe weight loss compared to 38.5%, pre-operatively while only 38.5% had BMI >23 kg/m^2^ compared to 84.6%, pre-operatively.

Moraes et al. [12] showed weight loss in more than half of patients after ileostomy who in the majority were above 50 years old, female, married and of incomplete elementary school. A relevant study conducted by Kim et al. [21] showed severe weight loss and BMI reduction, post-operatively among 72.7% (*n* = 50) of patients who underwent a colostomy or prophylactic ileostomy. Moreover, a weight loss of 5.2  ±  2.3 kg was present in 28% of stoma patients readmitted to hospital [22]. A reduction of BMI may be developed up to 40 days after hospital discharge [21] while a sharper BMI decrease is more prevalent in patients with high-output stoma (HOS) [6,22,23]. Early HOS (within 3 weeks of stoma formation) occurred in 75 (16%) of ileostomies/jejunostomies [23]. However, in less than two years after surgery, patients present adequate BMI [12].

The result of the current study that patients reduced the number of full meals and the intake of fruits and vegetables post-operatively is in line with Oliviera et al. [24] who showed that ileostomy patients (20%) avoided foods for fear of appliance leakage when compared with colostomy ones (4.8%), and reported the intake of vegetables and fruits as the most problematic. Interestingly, patients with an ileostomy tend to decrease total intake and restrict consumption of some foods due to repercussions on the volume and appearance of feces and other issues associated with aesthetics and well-being. Avoidance of certain foods may in turn increase the risk for nutritional deficiencies [12]. Notably, quality and quantity of food is crucial for ileostomy patients since reduction in protein intake may affect tissue repair after surgical construction of a stoma. Post-operatively, it is important to provide a high-energy, high-protein diet for wound healing that is low in excess insoluble fiber while pre-operatively, fiber and lactose intolerances are common [25]. Nutritional prehabilitation before major surgery is a matter of vital importance as it is shown to reduce post-operative complications, increase recovery speed, and improve patients’ quality of life. Noteworthy, prehabilitation is defined as the process of expanding patient’s functional and psychological capacity to reduce potential deleterious effects of a significant stressor, such as a surgical procedure and furthermore, it involves a multifactorial and interdisciplinary approach. Malnourished surgical patients have higher post-operative morbidity, mortality, length of hospital stay and readmission rates [26,27].

Messaris et al. [28] showed a 60-day readmission rate of 16.9% (*n* = 102) after colon or rectal resection with diverting loop ileostomy. Kulaylat et al. [29] showed creation of an ileostomy as an independent predictor for readmission within 30 days after a colectomy. Taking into consideration these elevated rates, it is easily to understand that malnutrition is an additional risk for complications. Migdanis et al. [30] recommend that an oral isotonic drink post discharge can have a prophylactic effect on patients with a newly formed ileostomy, preventing readmissions. It should be stressed that after hospital discharge, nutritional requirements may vary greatly depending on the remaining bowel, the fluid and electrolyte abnormalities, the overall health and other diagnoses [25].

Equally important is the finding of reduced mobility and independence of living in post surgery period. Indeed, patients experience physical impairment, deranged body function and emotional trauma, which further minimize their ability for self care and limit their social or sexual life [31,32,33,34]. Ang et al. [32] demonstrated that following ileostomy surgery, the most common stressors reported by patients during hospitalization included stoma formation, diagnosis of cancer, and preparation for self-care. After discharge, the stressors encompass adapting to body changes, altered sexuality, and impact on social life and activities. Self-efficacy plays an important role in the likelihood of adopting health behaviour changes and is associated with heightened motivation, treatment adherence and improved clinical and social outcomes [31,32,33,34]. Pre-and post-operative education in clinical settings regarding recovery process may be an essential step for patients and caregivers to cope with stoma stressors. Reinwalds et al. [33] indicated the following themes after an ileostomy: life being controlled by the altered bowel function, uncertainty regarding bowel function, and being limited in social life.

## 5. Limitations of the Study

This study has some limitations. Convenience sampling is one of the limitations in this study. This method is not representative of all population with an ileostomy living in Greece, thus limiting the generalizability of results.

Additionally, this was a pilot study which had the purpose to examine the feasibility of an approach that is intended to be used in a larger scale study. Furthermore, there were no blood tests along with nutritional assessment.

Given that it was a pilot study, the sample size was small and there was no sample size calculation, despite many significant associations being observed.

The strengths of the study include the wide spread instrument of MNA that may permit comparison among populations with an ileostomy.

Also, this pilot study involves 3 measurements with available pre-operative data since many studies do enroll patients after the construction of ileostomy.

## 6. Conclusions

This pilot study showed that in the 20th post-operative day, ileostomy patients had weight loss, reduced BMI, limited mobility, decrease in number of full meals, fruits and vegetables and less arm circumference.

Evaluation of baseline nutritional status of patients with colon cancer should be a part of routine clinical practice.

The understanding that nutritional deficit frequently accompanies an ileostomy, underpins the value of periodic nutritional assessment along with dietary education.

A multidisciplinary team of surgeons, nurses, gastroenterologists, nutritionists and hospital pharmacists needs to be established under the umbrella of a specially designed protocol for such cases.

Nutritional assessment as the most significant concern for people with an ileostomy should arguably be among research priorities.

It is anticipated that the present results will contribute to further research into this lifesaving procedure.

## Figures and Tables

**Table 1:** Distribution of sample according to demographic characteristic (*n* = 13).

Demographic characteristics	Median (IQR)
Age	62 (52.5–71)
Height (cm)	168.0 (164.5–173.0)
Weight	75.0 (66.5–83.5)
Body Mass Index BMI (kg/m^2^)	23.1 (16.7–29.9)

IQR – interquartile range.

**Table 2:** Mini Nutritional Assessment (MNA) score (time 0, 1, 2).

MNA Score	Median (IQR)
Mini Nutritional Assessment, pre-surgery (time 0)	24 (20.7–25.5)
Mini Nutritional Assessment, 7th post surgery (time 1)	18.5 (15–21.7)
Mini Nutritional Assessment, 20th post surgery (time 2)	19.0 (15.0–21.7)

**Table 3:** Factors associated with MNA (*n* = 13).

Variables	MNA Pre-Operatively			MNA 7th Post-Operatively			MNA 20th Post-Operatively			*p* Value
**Decrease in food intake**	***Severe***	***Moderate***	***No***	***Severe***	***Moderate***	***No***	***Severe***	***Moderate***	***No***	
	7.7	46.2	46.2	7.7	46.2	46.2	15.4	38.5	46.2	0.250
***Weight loss***	***>3 kg***	***1–3 kg***	***No***	***>3 kg***	***1–3 kg***	***No***	***>3 kg***	***1–3 kg***	***No***	
	38.5	23.1	38.5	46.2	15.4	38.5	53.8	15.4	30.8	**0.012**
**Mobility**	***Bad***	***Able***	***Goes out***	***Bad***	***Able***	***Goes out***	***Bad***	***Able***	***Goes out***	
		7.7	92.3	7.7	15.4	76.9	7.7	23.1	69.2	**0.023**
**Stress**	***Yes***	***No***		***Yes***	***No***		***Yes***	***No***		
	*38.5*	*61.5*		*46.2*	*53.8*		*46.2*	*53.8*		0.366
**Neuropsychological problems**	***Severe***	***Mild***	***No***	***Severe***	***Mild***	***No***	***Severe***	***Mild***	***No***	
	*7.7*	*92.3*		*7.7*	*92.3*		*7.7*	*92.3*		0.224
**BMI kg/m^2^**	***<19***	***19–23***	***>23***	***<19***	***19–23***	***>23***	***<19***	***19–23***	***>23***	
		15.4	84.6		38.5	61.5	7.7	61.5	30.8	**0.011**
**Independently life**	***Yes***	***No***		***Yes***	***No***		***Yes***	***No***		
	23.1	76.9			100		38.5	61.5		0.123
**>3 pills/per day**	***Yes***	***No***		***Yes***	***No***		***Yes***	***No***		
	30.8	69.2		30.8	69.2		38.5	61.5		0.213
**Pressure or skin ulcers**	***Yes***	***No***		***Yes***	***No***		***Yes***	***No***		
		100		15.4	84.6		15.4	84.6		0.111
Full meals	***1***	***2***	***3***	***1***	***2***	***3***	***1***	***2***	***3***	
	7.7	84.6	7.7	38.5	46.2	15.4	30.8	69.2		**0.023**
**>2 consumption fruit -vegetables**	***Yes***	***No***		***Yes***	***No***		***Yes***	***No***		
	*30.8*	*69.2.*			*100*			*100*		**0.007**
**Fluid consumption**	***>5***	***3–5***	***<3***	***>5***	***3–5***	***<3***	***>5***	***3–5***	***<3***	
	*69.2*	*30.8*		*15.4*	*46.2*	*38.5*	*53.8*	*38.5*	*7.7*	**0.024**
**Mode of feeding**	***Unable***	***Difficulty***	***No***	***Unable***	***Difficulty***	***No***	***Unable***	***Difficulty***	***No***	
			100	7.7	38.5	53.8	7.7	92.3		
**Arm circumference**	***<21***	***21–22***	***>22***	***<21***	***21–22***	***>22***	***<21***	***21–22***	***>22***	
			100	7.7	7.7	84.6	23.1	38.5	38.5	**0.002**
**Calf circumference**	***<31 cm***	***>31 cm***		***<31 cm***	***>31 cm***		***<31 cm***	***>31 cm***		
		100		7.7	92.3		30.8	69.2		**0.003**

Bold *p* trend values indicate a significance of *p* < 0.05.
